# Top Food Category Contributors to Sodium and Potassium Intake — United States, 2015–2016

**DOI:** 10.15585/mmwr.mm6932a3

**Published:** 2020-08-14

**Authors:** Rebecca C. Woodruff, Lixia Zhao, Jaspreet K.C. Ahuja, Cathleen Gillespie, Joseph Goldman, Diane M. Harris, Sandra L. Jackson, Alanna Moshfegh, Donna Rhodes, Rhonda S. Sebastian, Ana Terry, Mary E. Cogswell

**Affiliations:** ^1^Epidemic Intelligence Service, CDC; ^2^Division for Heart Disease and Stroke Prevention, National Center for Chronic Disease Prevention and Health Promotion, CDC; ^3^Beltsville Human Nutrition Research Center, Agricultural Research Service, U.S. Department of Agriculture; ^4^Division of Nutrition, Physical Activity and Obesity, National Center for Chronic Disease Prevention and Health Promotion, CDC; ^5^Division of Health and Nutrition Examination Surveys, National Center for Health Statistics, CDC.

Most U.S. adults consume too much sodium and not enough potassium (*1*,*2*). For apparently healthy U.S. adults aged ≥19 years, guidelines recommend reducing sodium intake that exceeds 2,300 mg/day and consuming at least 3,400 mg/day of potassium for males and at least 2,600 mg/day for females* (*1*). Reducing population-level sodium intake can reduce blood pressure and prevent cardiovascular diseases, the leading causes of death in the United States (*1*,*3*). Adequate potassium intake might offset the hypertensive effects of excessive sodium intake (*1*). Data from the 2015–2016 What We Eat in America (WWEIA) dietary interview component of the National Health and Nutrition Examination Survey (NHANES)^†^ were analyzed to identify top food categories contributing to sodium and potassium intake for U.S. residents aged ≥1 year. During 2015–2016, 40% of sodium consumed came from the top 10 food categories, which included prepared foods with sodium added (e.g., deli meat sandwiches and pizza). Approximately 43% of potassium consumed was from 10 food categories, which included foods naturally low in sodium (e.g., unflavored milk, fruit, vegetables) and prepared foods. These results can inform efforts to encourage consumption of foods naturally low in sodium, which might have the dual benefit of reducing sodium intake and increasing potassium intake, contributing to cardiovascular disease prevention.

This analysis used data from the 2015–2016 NHANES, a nationally representative survey and physical examination of the U.S. civilian, noninstitutionalized population. NHANES uses a multistage probability sampling design with oversampling of some age, race, and ethnicity (Hispanic or non-Hispanic). Among 9,544 participants (58.7% unweighted response rate), 7,976 (83.6%) were included in this analysis. Respondents were excluded if they were aged <1 year (4.0%), had an incomplete or unreliable initial 24-hour dietary recall or reported no energy intake (i.e., 0 kcal/day; 16.5%), consumed any human milk on the day of the recall (1.8%), were pregnant or had unknown pregnancy status (1.6%), or were lactating (0.5%).

The first of two, nonconsecutive, 24-hour dietary recalls was used for this analysis. The NHANES participant or a proxy completed the recall in person with trained interviewers using the U.S. Department of Agriculture’s automated multiple-pass method,^§^ which is designed to enhance complete and accurate food recall. Components of each reported food or beverage were assigned to mutually exclusive food codes with corresponding nutrient profiles, which provide the energy (in kilocalories) and nutrient content per 100 g.^¶^ Respondent total daily energy and sodium and potassium intake were calculated by summing the amount of each food consumed (in grams), multiplied by its assigned food code values. Sodium and potassium density were defined as milligrams of each nutrient per 1,000 kcal. For this analysis, food codes were grouped into 87 mutually exclusive categories adapted from the WWEIA Food Categories** (Supplementary Table, https://stacks.cdc.gov/view/cdc/91457) and included sandwiches and burgers as consumed (e.g., cheese sandwich rather than bread and cheese separately). Sodium included salt added in food preparation but did not include salt added at the table.

Data were analyzed with SAS-callable SUDAAN (version 9.3; RTI International) using day one dietary sample weights and accounting for the complex sampling design. The population proportions (*4*) of intake for each food category were estimated and ranked overall, and by age group (*1*), sex, race, ethnicity (Hispanic or non-Hispanic), and, for adults aged ≥19 years, by hypertension status (using the 2017 American College of Cardiology/American Heart Association [ACC/AHA] guideline)^††^ and weight status.^§§^

Among the U.S. population aged ≥1 year, mean sodium intake was 3,397 mg/day (Table 1). For that population, 40% of sodium was consumed from the top 10 food categories: deli meat sandwiches (6.3%), pizza (5.4%), burritos and tacos (5.3%), soups (4.1%), savory snacks (e.g., chips, crackers, popcorn) (3.8%), poultry (excluding nuggets and tenders) (3.7%), pasta mixed dishes (excluding macaroni and cheese, 3.0%), vegetables (excluding white potatoes) (2.9%), burgers (2.8%), and eggs and omelets (2.7%). The top five food categories contributing to sodium intake for almost all population subgroups (age [Table 1]; sex, race and Hispanic ethnicity [Table 2]; and blood pressure status and weight status [Table 3]) were among the top 10 categories for the U.S. population overall. Exceptions included unflavored milk (7.5%) among children aged 1–3 years; breads, rolls, and buns (4.0%) among adults aged ≥71 years; rice (9.0%) and breads, rolls, and buns (3.6%) among non-Hispanic Asians; quesadillas, tamales, fajitas, and enchiladas (4.3%) among Hispanics; and other meat sandwiches (3.6%) among adults aged ≥19 years with elevated blood pressure.

**TABLE 1:** Top 10 food category contributors (%)* to sodium and potassium intake, by age group — United States, 2015–2016

Food category and dietary contribution^†^	Proportion (%) in diet, by age group (yrs)								
	All age groups (≥1 year)	1–3	4–8	9–13	14–18	19–30	31–50	51–70	≥71
	(N = 7,976)	(n = 561)	(n = 828)	(n = 829)	(n = 756)	(n = 963)	(n = 1,617)	(n = 1,669)	(n = 753)
**Sodium^§^**									
1. Deli meat sandwiches	6.3	2.7	5.9	5.6	7.0	5.9	6.7	6.5	6.4
2. Pizza	5.4	5.1	6.9	7.7	10.7	6.8	4.5	3.8	—^¶^
3. Burritos and tacos	5.3	2.3	3.8	4.7	4.9	5.9	7.1	4.0	—^¶^
4. Soups	4.1	4.2	3.8	3.3	2.5	3.0	4.0	5.3	5.2
5. Savory snacks (e.g., chips, crackers, popcorn)	3.8	7.2	6.8	5.9	4.2	3.7	3.0	3.4	2.9
6. Poultry (excl. nuggets and tenders)	3.7	3.4	2.5	2.6	3.2	5.2	4.1	3.1	2.4
7. Pasta mixed dishes (excluding macaroni and cheese)	3.0	4.0	3.1	3.5	2.9	3.5	2.8	2.3	3.6
8. Vegetables (excluding white potatoes)	2.9	3.0	1.9	1.6	1.6	2.3	3.0	4.0	4.0
9. Burgers	2.8	—^¶^	2.7	2.5	4.2	2.7	3.0	2.7	1.4
10. Eggs and omelets	2.7	3.7	2.2	2.0	1.7	3.3	2.6	2.5	3.2
**Total contribution of top 10 food categories**	**40.0**	**35.6**	**39.6**	**39.4**	**42.9**	**42.3**	**40.8**	**37.6**	**29.1**
Mean daily sodium intake (mg) (SE)	3,397 (34)	1,929 (58)	2,655 (43)	3,260 (62)	3,423 (90)	3,861 (98)	3,722 (68)	3,431 (46)	2,861 (88)
Mean daily sodium density (mg/1,000 kcal) (SE)	1,692 (12)	1,465 (25)	1,549 (14)	1,663 (24)	1,689 (25)	1742 (32)	1,726 (22)	1,723 (20)	1,653 (32)
Potassium**									
1. Milk, unflavored	6.4	24.8	13.1	10.0	8.9	4.2	4.6	5.0	7.1
2. Fruit	6.4	10.3	8.0	6.5	5.9	5.6	5.3	6.6	8.5
3. Vegetables (excluding white potatoes)	6.1	3.3	3.6	3.5	2.9	5.5	6.6	7.6	7.5
4. Coffee	5.1	0.0	0.1	—^¶^	0.9	3.7	5.6	8.1	7.1
5. Savory snacks (e.g., chips, crackers, popcorn)	3.5	3.8	5.1	5.4	4.6	3.3	3.4	3.0	2.3
6. 100% fruit juice	3.3	7.7	5.3	4.0	4.0	3.9	2.7	2.2	3.5
7. Mashed, baked, or boiled white potatoes	3.2	1.9	1.8	3.2	2.8	3.0	3.0	3.5	4.3
8. Deli meat sandwiches	3.1	1.1	3.0	3.1	4.0	3.4	3.6	2.8	2.8
9. Poultry (excluding nuggets and tenders)	2.9	2.2	2.0	2.0	2.6	4.4	3.4	2.4	1.7
10. Burritos and tacos	2.9	1.0	2.1	2.8	2.9	3.5	3.8	2.2	—^¶^
**Total contribution of top 10 food categories**	**42.9**	**56.1**	**44.1**	**40.5**	**39.5**	**40.5**	**42.0**	**43.4**	**44.8**
Mean daily potassium intake (mg) (SE)	2,497 (35)	1,797 (35)	1,968 (52)	2,168 (52)	2,235 (60)	2,581 (62)	2,675 (72)	2,708 (41)	2,401 (77)
Mean daily potassium density (mg/1,000 kcal) (SE)	1,276 (12)	1,395 (19)	1,175 (21)	1,113 (15)	1,127 (18)	1,190 (20)	1,266 (16)	1,387 (18)	1,401 (31)
Mean daily energy intake (kcal) (SE)	2,041 (18)	1,321 (29)	1,714 (27)	1,995 (38)	2,062 (54)	2,262 (47)	2,211 (35)	2,039 (24)	1,760 (44)

**Abbreviations:** kcal = kilocalories; SE = standard error.

* The population proportion (%) is defined as the sum of the amount of sodium or potassium consumed from each specific food category for all participants in the designated group, divided by the sum of the nutrient consumed from all food categories for all participants in the designated group, multiplied by 100. All estimates use one 24-hour dietary recall, reflect the complex sampling design, and use the day one dietary sample weights to account for nonresponse, weekend/weekday recalls, and oversampling.

^†^ This analysis used 87 food categories, which were adapted from What We Eat in America (https://www.cdc.gov/nchs/nhanes/wweia.htm). Food categories are ranked in descending order by population proportion among the total population aged ≥1 year.

^§^ The following food categories were not ranked among the top 10 sodium food sources overall but contributed ≥3% to sodium intake within the specified age subgroups: 1–3 years: unflavored milk (7.5%), cheese (3.7%), bacon, frankfurters, sausages (3.3%), chicken patties, nuggets, and tenders (3.0%); 4–8 years: bacon, frankfurters, sausages (3.3%), hot dog and sausage sandwiches (3.3%), unflavored milk (3.1%), cookies, brownies, cakes (3.1%), ready to eat cereals (3.0%); 14–18 years: chicken patties, nuggets, and tenders (3.2%); 51–70 years: meat mixed dishes (3.4%), breads, rolls, buns (3.0%); ≥71 years: breads, rolls, buns (4.0%), cookies, brownies, cakes (4.0%).

^¶^ Estimates are statistically unreliable, relative SE ≥30%.

** The following food categories were not ranked among the top 10 potassium food sources overall but contributed ≥3% of potassium intake within the specified age subgroups: 4–8 years: flavored milk (7.0%), pizza (3.4%); 9–13 years: flavored milk (4.2%), pizza (4.0%), pasta mixed dishes (excluding macaroni and cheese (3.0%); 14–18 years: pizza (5.7%), fried white potatoes (3.5%), burgers (3.0%); 19–30 years: fried white potatoes (3.6%), pizza (3.2%); 31–50 years: fried white potatoes (3.2%), alcoholic beverages (3.1%).

**TABLE 2:** Top 10 food category contributors (%)* to sodium and potassium, by race/ethnicity — United States, 2015–2016

Food category and dietary contribution^†^	Proportion (%) in diet, by sex		Proportion (%) in diet, by race/ethnicity			
	Male	Female	White, non-Hispanic	Black, non-Hispanic	Asian, non-Hispanic	Hispanic
	(n = 3,984)	(n = 3,992)	(n = 2,578)	(n = 1,727)	(n = 743)	(n = 2,537)
**Sodium^§^**						
1. Deli meat sandwiches	7.1	5.2	7.6	4.6	2.4	4.2
2. Pizza	6.0	4.6	5.5	5.5	3.2	6.0
3. Burritos and tacos	5.7	4.6	4.6	2.1	1.8	11.6
4. Soups	3.7	4.6	3.1	3.1	12.8	4.5
5. Savory snacks (e.g., chips, crackers, popcorn)	3.6	4.1	4.0	4.3	2.6	3.2
6. Poultry (excluding nuggets and tenders)	3.9	3.3	2.7	7.9	4.0	4.1
7. Pasta mixed dishes (excluding macaroni and cheese)	3.2	2.7	3.3	3.6	2.1	1.8
8. Vegetables (excluding white potatoes)	2.5	3.5	2.9	3.3	5.4	1.8
9. Burgers	3.2	2.1	2.9	3.3	0.6	2.7
10. Eggs and omelets	2.5	2.9	2.6	2.2	2.5	3.6
**Total contribution of top 10 food categories**	**41.4**	**37.6**	**39.2**	**39.9**	**37.4**	**43.5**
Daily sodium intake (mg) (SE)	3,871 (56)	2,927 (32)	3,404 (49)	3,258 (59)	3,710 (100)	3,332 (47)
Daily sodium density (mg/1,000 kcal) (SE)	1,701 (19)	1,683 (16)	1,685 (16)	1,645 (18)	2,020 (75)	1,640 (17)
**Potassium^¶^**						
1. Milk, unflavored	6.7	6.2	6.5	4.9	5.9	
2. Fruit	5.8	7.1	6.1	5.7	9.9	6.7
3. Vegetables (excluding white potatoes)	5.0	7.5	6.1	5.9	9.5	5.0
4. Coffee	5.2	5.0	6.4	2.0	2.5	3.2
5. Savory snacks (e.g., chips, crackers, popcorn)	3.5	3.4	3.5	5.0	2.6	2.8
6. 100% fruit juice	3.4	3.1	2.7	5.1	3.0	4.7
7. Mashed, baked or boiled white potatoes	2.9	3.5	3.7	2.9	1.0	2.1
8. Deli meat sandwiches	3.6	2.5	3.7	2.5	1.3	2.3
9. Burritos and tacos	3.2	2.5	2.5	1.3	1.2	6.0
10. Poultry (excluding nuggets and tenders)	3.1	2.6	2.3	5.4	3.2	3.5
**Total contribution of top 10 food categories**	**42.4**	**43.4**	**43.5**	**40.7**	**40.1**	**43.4**
Mean daily potassium intake (mg) (SE)	2,771 (39)	2,225 (39)	2,563 (42)	2,191 (44)	2,423 (41)	2,590 (58)
Mean daily potassium density (mg/1,000 kcal) (SE)	1,237 (12)	1,315 (18)	1,297 (15)	1,149 (16)	1,405 (17)	1,252 (13)
Mean daily energy intake (kcal) (SE)	2,318 (27)	1,765 (12)	2,059 (21)	1,995 (33)	1,902 (40)	2,033 (24)

**Abbreviations:** kcal = kilocalories; SE = standard error.

* The population proportion (%) is defined as the sum of the amount of sodium or potassium consumed from each specific food category for all participants in the designated group, divided by the sum of the nutrient consumed from all food categories for all participants in the designated group, multiplied by 100. All estimates use one 24-hour dietary recall, reflect the complex sampling design, and use the day one dietary sample weights to account for nonresponse, weekend/weekday recalls, and oversampling.

^†^ This analysis used 87 food categories, which were adapted from What We Eat in America (https://www.cdc.gov/nchs/nhanes/wweia.htm). Food categories are ranked in descending order by population proportion among the total population aged ≥1 year.

^§^ The following food categories were not ranked among the top 10 sodium food sources overall but contributed ≥3% to overall sodium intake. Non-Hispanic Asians: rice (9.0%); breads, rolls, buns (3.6%); soy-based condiments (3.2%); fried rice and lo/chow mein (3.1%). Hispanics: quesadillas, tamales, fajitas and enchiladas (4.3%).

^¶^ The following food categories were not ranked among the top 10 potassium food sources overall but contributed ≥3% to overall potassium intake among sex and race/ethnic subgroups. Non-Hispanic blacks: fried white potatoes (4.3%); soft drinks, fruit drinks, and sport/energy drinks (3.1%). Non-Hispanic Asians: soups (6.5%). Hispanics: beans, peas, legumes (4.0%); soups (3.0%).

**TABLE 3:** Top 10 food category contributors (%)* to sodium and potassium intake, by blood pressure status and weight status among adults aged ≥19 years – United States, 2015–2016

Food category and dietary contribution^†^	Proportion (%) in diet, total	Proportion (%) in diet, by blood pressure status^§^			Proportion (%) in diet, by weight status^¶^		
		Normal blood pressure	Elevated blood pressure	Stage I or II hypertension	Normal weight and underweight	Overweight	Obesity
	(N = 5,002)	(n = 1,713)	(n = 667)	(n = 2,546)	(n = 1,344)	(n = 1,596)	(n = 2,015)
Sodium**							
1. Deli meat sandwiches	6.5	6.8	6.0	6.4	5.3	7.0	6.8
2. Burritos and tacos	5.5	5.5	5.4	5.6	4.0	5.8	6.4
3. Pizza	4.6	5.3	3.7	4.3	5.4	3.6	5.1
4. Soups	4.3	4.6	3.5	4.3	5.5	3.9	3.8
5. Poultry (excluding nuggets and tenders)	3.9	4.1	5.1	3.3	4.2	4.0	3.5
6. Savory snacks (e.g., chips, crackers, popcorn)	3.3	3.6	3.2	3.2	3.2	3.5	3.3
7. Vegetables (excluding white potatoes)	3.2	3.1	3.3	3.3	3.5	3.4	2.9
8. Pasta mixed dishes (excluding macaroni and cheese)	2.9	2.5	2.7	3.3	3.3	2.8	2.7
9. Eggs and omelets	2.8	2.9	3.0	2.6	2.8	3.3	2.4
10. Burgers	2.7	2.8	2.3	2.7	2.6	2.9	2.6
**Total contribution of top 10 food categories**	**39.7**	**41.1**	**38.2**	**39.0**	**39.6**	**40.2**	**39.5**
Mean daily sodium intake (mg) (SE)	3,545 (42)	3,573 (61)	3,699 (61)	3,472 (66)	3,528 (58)	3,477 (72)	3,614 (86)
Mean daily sodium density (mg/1,000 kcal) (SE)	1,718 (14)	1,726 (22)	1,710 (38)	1,715 (19)	1729 (28)	1,671 (20)	1,750 (27)
Potassium^††^							
1. Vegetables (excluding white potatoes)	6.8	7.1	7.0	6.6	7.7	6.9	6.1
2. Coffee	6.3	5.6	7.0	6.7	5.8	6.6	6.4
3. Fruit	6.2	6.6	5.6	6.0	7.4	6.2	5.3
4. Milk, unflavored	4.9	4.4	5.4	5.2	5.7	4.9	4.5
5. Mashed, baked or boiled white potatoes	3.3	2.9	3.1	3.8	3.1	2.8	3.9
6. Deli meat sandwiches	3.2	3.4	3.1	3.1	2.6	3.5	3.4
7. Savory snacks (e.g., chips, crackers, popcorn)	3.1	3.3	2.8	3.1	2.8	3.5	3.1
8. Poultry (excluding nuggets and tenders)	3.1	3.4	4.0	2.6	3.0	3.3	2.9
9. Burritos and tacos	3.0	3.0	2.9	3.0	2.2	2.9	3.6
10. 100% fruit juice	2.9	3.0	2.9	2.8	3.5	2.7	2.6
**Total contribution of top 10 food categories**	**42.9**	**42.7**	**43.8**	**42.8**	**43.6**	**43.4**	**41.8**
Mean daily potassium intake (mg) (SE)	2,636 (39)	2,643 (57)	2,719 (67)	2,603 (43)	2,694 (57)	2,656 (66)	2,566 (45)
Mean daily potassium density (mg/1,000 kcal) (SE)	1,308 (14)	1,308 (22)	1,280 (20)	1,317 (17)	1,342 (24)	1,315 (20)	1,274 (16)
Mean daily energy intake (kcal) (SE)	2,108 (21)	2,114 (23)	2,197 (45)	2,075 (33)	2,099 (32)	2,109 (41)	2,113 (27)

**Abbreviations:** BMI = body mass index; BP = blood pressure; kcal = kilocalories; SE = standard error.

* The population proportion (%) is defined as the sum of the amount of sodium or potassium consumed from each specific food category for all participants in the designated group, divided by the sum of the nutrient consumed from all food categories for all participants in the designated group, multiplied by 100. All estimates use one 24–hour dietary recall, reflect the complex sampling design, and use the day one dietary sample weights to account for nonresponse, weekend/weekday recalls, and oversampling.

^†^ This analysis used 87 food categories, which were adapted from What We Eat in America (https://www.cdc.gov/nchs/nhanes/wweia.htm). Food categories are ranked in descending order by population proportion among the total population aged ≥1 year.

^§^ Blood pressure was defined as normal (<120/80 mmHg), elevated (systolic BP = 120–129 and diastolic BP <80 mmHg), or stage I or II hypertension (self–reported antihypertensive medication use or systolic BP ≥130, diastolic BP ≥80 mmHg) according to the 2017 American College of Cardiology/American Heart Association Hypertension Guideline.

^¶^ BMI (kg/m^2^) was used to classify adults: normal weight (BMI = 18.5–24.9), overweight (BMI = 25.0–29.9), or obesity (BMI ≥30).

** The following food categories were not ranked as top 10 sodium food sources among respondents aged ≥19 years but contributed ≥3% to sodium intake among blood pressure and weight status subgroups. Elevated BP: other meat sandwiches (3.6%), poultry sandwiches (3.0%). Stage I or II hypertension: meat mixed dishes (3.1%).

^††^ The following food categories were not ranked as top 10 potassium food sources among respondents aged ≥19 years but contributed ≥3% to potassium intake among blood pressure and weight status subgroups. Obese: fried white potatoes (3.5%).

Mean potassium intake was 2,497 mg/day overall (Table 1). Overall, 43% of potassium was consumed from the top 10 food categories: unflavored milk (6.4%); fruit (6.4%); vegetables (excluding white potatoes) (6.1%); coffee (5.1%); savory snacks (e.g., chips, crackers, popcorn) (3.5%); 100% fruit juice (3.3%); mashed, baked, or boiled white potatoes (3.2%); deli meat sandwiches (3.1%); poultry (excluding nuggets and tenders) (2.9%); and burritos and tacos (2.9%). These food categories contributed varying amounts to total potassium intake by age subgroup, ranging from 39.5% among youths aged 14–18 years to 56.1% among children aged 1–3 years. For almost all population subgroups, the top five food categories contributing to potassium intake were among the top 10 categories for the overall population (Tables 1, 2, and 3). Exceptions included flavored milk among children aged 4–8 years (7.0%) and 9–13 years (4.2%), pizza among youth aged 14–18 years (5.7%), and soups among non-Hispanic Asians (6.5%).

## Discussion

This analysis found that 40% of sodium intake and 43% of potassium intake came from the top 10 food categories for each nutrient. The analysis provides the most current information about the top food categories contributing to sodium and potassium intake in the United States. Consistent with prior analyses (*5*,*6*), the top contributors to sodium intake primarily included prepared foods with sodium added (e.g., deli meat sandwiches, poultry, or vegetables with added sodium). As indicated in earlier research (*7*), potassium intake primarily comes from foods that are naturally low in sodium (e.g., unflavored milk, fruit, and vegetables) and prepared foods. Notably, five food categories (deli meat sandwiches, burritos and tacos, savory snacks, poultry, and vegetables) ranked as top 10 contributors for sodium and potassium intake overall, highlighting the interconnected nature of the food categories contributing to intake of both nutrients.

Multiple federal agencies have ongoing initiatives promoting the National Academies of Medicine (formerly Institute of Medicine) recommendations for sodium reduction and the expansion of healthier food options (*8*). For example, the Food and Drug Administration developed draft guidance on voluntary targets for sodium added to the U.S. food supply.^¶¶^ In addition, the Food Service Guidelines for Federal Facilities*** expand access to healthy food options and can be adapted for use in hospitals, government facilities, afterschool and recreational programs, faith-based organizations, and other institutions. CDC programs that fund the implementation of food service guidelines include the Sodium Reduction in Communities,^†††^ State Physical Activity and Nutrition,^§§§^ and High Obesity programs.^¶¶¶^

The findings in this report are subject to at least three limitations. First, dietary data were self-reported and are susceptible to recall and social desirability biases. The automated multiple-pass method might underestimate sodium and potassium intake (*9*). Estimates do not include sodium from salt added at the table, which might contribute 5%–6% to total intake (*10*), or potassium from supplements. Second, the 2017 ACC/AHA guidelines redefining hypertension had not been released at the time of data collection. Some persons classified as having hypertension in this analysis might have been unaware of their change in status. However, this approach permitted assessment of food categories contributing to intake in relation to the current definition of hypertension, which can inform public health strategies to reduce cardiovascular disease risk. Finally, differences in the top food categories reported in this analysis as compared with prior analyses (*5*–*7*) might be attributable to variation in how foods were categorized, rather than to changes in consumer behavior. This analysis counted sandwich toppings or other additions (e.g., condiments) as part of the sandwich categories, but other foods consumed in combination (e.g., salads) were treated as separate food categories (e.g., lettuce, salad dressing), which might have resulted in sandwiches being more likely to be ranked among the top food categories as compared with other foods consumed in combination.

Monitoring the food categories contributing to population sodium and potassium intake can inform cardiovascular disease prevention initiatives. Consuming foods naturally low in sodium (e.g., fruits and vegetables without added sodium) in place of foods that are high in sodium (e.g., prepared foods with added sodium) might have the dual benefit of decreasing sodium intake and increasing potassium intake (*1*). In addition, differences in top food categories contributing to sodium and potassium intake by race and Hispanic ethnicity indicate the need for dietary strategies that encompass the variability in foods consumed to reach populations at elevated risk for cardiovascular disease. Understanding the top food categories contributing to sodium and potassium intake informs individual and public health strategies to lower blood pressure and reduce cardiovascular disease risk.

**Summary**

**What is already known about this topic?**

Most U.S. residents consume too much sodium and too little potassium, increasing the risk for cardiovascular disease.

**What is added by this report?**

During 2015–2016, approximately 40% of sodium intake came from the top 10 food categories, which included prepared foods with added sodium (e.g., deli meat sandwiches, pizza, burritos and tacos, soups, savory snacks). Approximately 43% of potassium intake came from the top 10 categories, which included foods low in added sodium (e.g., unflavored milk, fruit, vegetables) and prepared foods.

**What are the implications for public health practice?**

Increasing intake of foods that are naturally low in added sodium (e.g., fruits and vegetables without added salt) might have the dual benefit of decreasing sodium intake and increasing potassium intake.

## Footnotes

* The 2015–2020 Dietary Guidelines for Americans recommends that persons in the United States limit their sodium intake. The 2019 Dietary Reference Intake values for sodium and potassium intake vary by age, with lower values for children than for adults.

† https://www.cdc.gov/nchs/nhanes/index.htm.

§ https://www.ars.usda.gov/northeast-area/beltsville-md-bhnrc/beltsville-human-nutrition-research-center/food-surveys-research-group/docs/ampm-usda-automated-multiple-pass-method/.

¶ https://www.ars.usda.gov/fsrg/fndds.

** https://www.ars.usda.gov/northeast-area/beltsville-md-bhnrc/beltsville-human-nutrition-research-center/food-surveys-research-group/docs/dmr-food-categories/.

†† Hypertension status was determined using the average of up to three blood pressure measurements collected during an examination and reported use of antihypertensive medication. The 2017 American College of Cardiology/American Heart Association Hypertension guideline uses the following classifications: 1) normal blood pressure: <120/80 mmHg, 2) elevated blood pressure: systolic 120–129 mmHg and diastolic <80mmHg, and 3) stage I or II hypertension: self-reported antihypertensive medication use or systolic ≥130 mmHg, diastolic ≥80 mmHg. https://www.ahajournals.org/doi/10.1161/HYP.0000000000000065.

§§ Weight status was categorized according to body mass index (BMI) (kg/m^2^), which was determined by measured heighted and weight: underweight or normal weight (BMI <25.0), overweight (BMI = 25.0–29.9), or obesity (BMI ≥30.0).

¶¶ https://www.fda.gov/regulatory-information/search-fda-guidance-documents/draft-guidance-industry-target-mean-and-upper-bound-concentrations-sodium-commercially-processed.

*** https://www.cdc.gov/obesity/downloads/guidelines_for_federal_concessions_and_vending_operations.pdf.

††† https://www.cdc.gov/dhdsp/programs/sodium_reduction.htm.

§§§ https://www.cdc.gov/nccdphp/dnpao/state-local-programs/span-1807/index.html.

¶¶¶ https://www.cdc.gov/nccdphp/dnpao/state-local-programs/hop-1809/high-obesity-program-1809.html.
